# Chronic *Prosopis Glandulosa* Treatment Blunts Neutrophil Infiltration and Enhances Muscle Repair after Contusion Injury

**DOI:** 10.3390/nu7020815

**Published:** 2015-01-23

**Authors:** Cindy George, Carine Smith, Ashwin W. Isaacs, Barbara Huisamen

**Affiliations:** 1 Department of Biomedical Sciences, Faculty of Health Science, Stellenbosch University, Tygerberg 7505, South Africa; E-Mail: bh3@sun.ac.za; 2 Department of Physiological Sciences, Faculty of Science, Stellenbosch University, Stellenbosch 7600, South Africa; E-Mails: csmith@sun.ac.za (C.S.); awisaacs@sun.ac.za (A.W.I.); 3 Medical Research Council, Diabetes Discovery Platform, Francie van Zijl Drive, Parowvallei, Tygerberg 7505, South Africa

**Keywords:** fabaceae, inflammation, mass-drop injury, NSAID, regeneration

## Abstract

The current treatment options for soft tissue injuries remain suboptimal and often result in delayed/incomplete recovery of damaged muscle. The current study aimed to evaluate the effects of oral *Prosopis glandulosa* treatment on inflammation and regeneration in skeletal muscle after contusion injury, in comparison to a conventional treatment. The gastrocnemius muscle of rats was subjected to mass-drop injury and muscle samples collected after 1-, 3 h, 1- and 7 days post-injury. Rats were treated with *P. glandulosa* (100 mg/kg/day) either for 8 weeks prior to injury (up until day 7 post-injury), only post-injury, or with topically applied diclofenac post-injury (0.57 mg/kg). Neutrophil (His48-positive) and macrophage (F4/80-positive) infiltration was assessed by means of immunohistochemistry. Indicators of muscle satellite cell proliferation (ADAM_12_) and regeneration (desmin) were used to evaluate muscle repair. Chronic *P. glandulosa* and diclofenac treatment (*p <* 0.0001) was associated with suppression of the neutrophil response to contusion injury, however only chronic *P. glandulosa* treatment facilitated more effective muscle recovery (increased ADAM_12_ (*p <* 0.05) and desmin (*p <* 0.001) expression), while diclofenac treatment had inhibitory effects on repair, despite effective inhibition of neutrophil response. Data indicates that *P. glandulosa* treatment results in more effective muscle repair after contusion.

## 1. Introduction

Soft tissue injuries are very common, accounting for between 35% and 55% of all sporting injuries [1]. Soft tissue injuries can result in significant pain, swelling and bruising, culminating in delayed and impaired functionality of the affected muscle [2]. The pathophysiology of muscle injuries is a complex process, progressing through a sequence of overlapping phases, which include degeneration, inflammation, regeneration and the formation of fibrotic scar tissue [3,4,5,6]. Injuries to skeletal muscle not only damages the muscle cells itself, but may also lead to capillary rupture, infiltrative bleeding, inflammation, oxidative stress and fibrosis, depending on the extent of the injury. Inflammation stands central to these processes, with inflammatory cytokines largely responsible for modulating the cellular environment, thereby largely controlling the progress of other repair processes. Therefore, inflammation is the most likely target to consider when developing treatments to facilitate faster recovery.

In recent years, researchers have focused on manipulation of inflammation to accelerate muscle regeneration, for example, by targeting immune cells activated during the inflammatory phase [7,8]. Neutrophils and macrophages enter the site of injury in response to chemotactic signals and phagocytize the local debris [5,9,10]. Neutrophils, along with macrophages and satellite cells, release oxygen free radicals, resulting in oxidative stress and direct damage to surrounding tissue unaffected by the primary injury, which results in secondary muscle damage. However, even though early stage phenotypes of macrophages partially contributes to the sustainability of the inflammatory response and thus also secondary damage, these cells also secrete various growth factors that directly contribute to tissue repair and regeneration [5,11]. Additionally, both neutrophils and macrophages stimulate the release of cytokines (IL-1, IL-6, IL-8) and other chemotactic factors by T-cells, which inevitably results in the recruitment of satellite cells, with thus a greater capacity for muscle regeneration [5,12,13]. It is therefore clear that the inflammatory response, even though a contributor to secondary damage, is crucial to the repair of skeletal muscle after injury. Thus in the event of this total process being severely blunted for a prolonged period of time, such as through non-steroidal anti-inflammatory drug (NSAID) treatment, the potential clinical outcome may be suboptimal, resulting in delayed and/or incomplete tissue healing, as well as excessive scar formation, which increases the risk for recurrence of injury. There is evidence that suggests that the prolonged inhibition of the cyclooxygenase-2 pathway (more than 7 days continually) with prostaglandin inhibitors (NSAIDs) compromises muscle repair [4,14,15]. In addition to the use of NSAID’s, there are various other muscle injury treatment options, such as the RICE approach (rest, ice, compression and elevation) [4,16,17,18], therapeutic ultrasound [19,20], hyperbaric oxygen therapy [21] and the use of growth factors [22]. However, these therapies remain suboptimal, as in many instances it either does not translate into increased myotube formation, therefore does not enhance muscle healing [19,20] and may be associated with severe risks and side effects [23].

The potential for targeting the inflammatory response using natural products was recently illustrated in a similar model to the one used in our study, when accelerated skeletal muscle recovery (after treatment with a grape-seed derived polyphenol) was associated with a suppression of the neutrophil response to contusion injury, less secondary damage and earlier macrophage phenotype switch which resolved inflammation faster [7,24]. We therefore hypothesized that treatment with the pods of *Prosopis glandulosa* (Torr.) (Fabaceae) would blunt neutrophil infiltration into the site of injury, thereby alleviating the inflammatory response and consequently enhance muscle regeneration. Mesquite, which is a common name for several species of the leguminous plants of the *Prosopis* genus, have been found to contain numerous phytochemicals eliciting various effects, such as anti-inflammatory effects [25]. Very few studies have been conducted on the *P. glandulosa* plant itself and no literature could be found regarding its potential clinical benefits, except those from our own laboratory [26,27], which were performed in a different context (diabetes and cardiovascular health). However, since diabetes and cardiovascular health both have known inflammatory components, it is likely that this product could also have therapeutic application in the context of inflammatory muscle injury. Particular strengths of this study are the inclusion of a recognized NSAID commonly used in the treatment of muscle injury and inflammation–diclofenac–as comparative control, as well as the fact that the treatment was tested for both its capacity as a preventative chronic supplement and for acute therapeutic application.

## 2. Experimental Section

### 2.1. Experimental Animals

Age- and weight-matched adult, male, Wistar rats were used. All animals were housed at the Stellenbosch University Central Research Facility, Tygerberg, in temperature controlled rooms (22–24 °C) and kept on a 12-h light/dark cycle (lights on at 6:30 am). Rats were given *ad libitum* access to standard laboratory rat chow pellets and tap water for the duration of the experimentation. The animals received humane care in accordance with the principles of the South African National Standard for the care and use of animals for scientific purposes (South African Bureau of Standards, SANS 10386, 2008). The project was approved by the Animal Research Ethics Committee of Sub-Committee B of Stellenbosch University (reference #10GK_HIL01).

Experimental rats were divided into four main groups, namely: (1) control placebo (PLA); (2) PG-CHR, animals treated with *P. glandulosa* for 8 weeks prior to injury and after injury, up to the time of sacrifice; (3) PG-AI, animals treated with *P. glandulosa* after injury (first treatment, 2 h after injury), up to the time of sacrifice and (4) NSAID, animals treated with Voltaren Emulgel^®^ (diclofenac) directly after injury, up to the time of sacrifice. All four groups were subdivided into sacrifice and data collection time points of *t* = 0 h (before injury), 1 h, 3 h, 1 day and 7 days, post-injury. Each main group had an *n* = 25, *i.e.*, 5 rats per time point per group (total of 100 rats).

Rats in the different experimental groups were matched for body mass at the start of the protocol (PLA: 456.47 ± 9.74 g; PG-CHR: 445.98 ± 11.21 g; PG-AI: 439.12 ± 14.84 g; NSAID: 442.25 ± 12.58 g).

### 2.2. P. Glandulosa and Diclofenac Treatments

The *P. glandulosa* powder consisted solely of dry-milled *P. glandulosa* pods [26]. To prepare treatment, *P. glandulosa* powder was weighed daily for each animal in the treatment group and set into a mixture of commercially available gelatine/jelly cubes of 1 mL volume. These jelly cubes were fed to each animal individually for 8 weeks, to ensure absolute compliance and dose control. The dosage of 100 mg/kg/day *P. glandulosa* was calculated based on the daily dosage prescribed for human adults on this commercially available supplement. We have previously shown this dose to elicit beneficial metabolic changes in rats [26,27]. During the 8-week experimental period, the control animals received placebo jelly cubes.

The *P. glandulosa* preparation was supplied by P. Schoeman, who is the patent holder of the *P. glandulosa* supplement preparation method. A voucher herbarium specimen was prepared and lodged at the Stellenbosch herbarium. The composition of the product was analyzed by the CSIR (SA), J. Muller Laboratories (Pty) Ltd. and the Central Analytical Facility of the Stellenbosch University and all three institutions concurred on the chemical content thereof. Addendum A contains an example of the chemical analysis of this proprietary drug, as analyzed by J. Muller Laboratories (Pty) Ltd.

Diclofenac, a known NSAID, served as a positive control for the anti-inflammatory effects of treatment. Diclofenac sodium*,* in the form of Voltaren Emulgel^®^, was applied topically to the injured area on the hindlimb of the rats after different time periods post-injury. The dosage of Voltaren Emulgel^®^ was calculated at 57.14 mg/kg/day, which equaled to 0.57 mg/kg Diclofenac. The dosage was calculated based on the daily dosage prescribed for human adults.

### 2.3. Induction of Experimental Muscle Contusion Injury and Sample Collection

The contusion injury to the rat hind-limb was produced using the mass-drop model injury first described by Stratton *et al.* (1984) [28] and optimized for our laboratory by Myburgh and colleagues (2012) [7]. Briefly, the technique entails dropping a 200 g weight from the height of 50 cm onto the medial surface of the right gastrocnemius muscle of sodium pentobarbital (40 mg/kg, intraperitoneal) anaesthetized rats. This contusion injury was moderately severe, did not result in bone injury or affect gait in the injured animals.

For sample collection, rats were euthanized by sodium pentobarbital overdose (200 mg/kg, intraperitoneal) and the central section of the damaged gastrocnemius muscle harvested. The harvested muscle was divided into two parts, one part processed for immunohistochemistry and the other part snap-frozen for Western blotting analysis.

### 2.4. Muscle Histology and Immunohistochemistry

For cross-sectional histology and immunohistochemistry, muscles were fixed in 10% formal saline, processed and embedded in paraffin wax. Five-micrometer thick cross-sections were prepared (Leica RM 2125 RT microtome, Nussloch, Germany) and stained with haematoxylin and eosin (H & E) for qualitative histological analysis.

Immunostaining with mouse anti-rat His48 (neutrophil; 1:200; Santa Cruz Biotechnology, Santa Cruz, CA, USA), goat anti-mouse F4/80 (macrophage; 1:200; Santa Cruz Biotechnology, Santa Cruz, CA, USA) and rabbit anti-human desmin (1:200; Santa Cruz Biotechnology, Santa Cruz, CA, USA) antibodies was performed on the fully automated Leica Bond-Max Autostainer system (Leica Microsystems, Germany) using an onboard detection kit, which included the Bond Epitope Retrieval Solution, peroxide block, primary antibody, post primary reagent, Bond Wash solution and Bond Polymer [29,30]. DAB (3,3′-diaminobenzidine tetrahydrochloride) was used as the chromogen (Leica Microsystems, Germany). Appropriate positive controls were used throughout the study.

### 2.5. Image Analysis

All imaging data were obtained by analyzing two sections from each muscle sample, at each time point for each antibody. In the injured area, five fields of view per section were imaged using a microscope (Nikon ECLIPSE E400; 400x objective used), equipped with a color digital camera (Nikon 5.0 Mega Pixels Color Digital Camera head DS-Fi2). The images presented in this article are only partial images of those taken at 200× magnification. Photos were used to count positively labeled neutrophils, macrophages and desmin-stains. Immune cells were counted manually and expressed as the average number of positively labeled immune cells per field of view (350 μm^2^) in the injured area, using the NIS-Elements BR imaging software package. In order for a cell to be classified a true neutrophil and macrophage, it had to have multilobular nuclei or single nuclei with surrounding cytoplasm, respectively.

### 2.6. Western Blotting

Protein levels were determined by standard Western blotting technique [26]. Briefly, proteins were extracted from the gastrocnemius muscle tissue, equal concentration of total protein loaded and separated on a SDS poly-acrylamide gel and electro-transferred to ImmobilonTM-P PVDF membranes. Ponceau red reversible stain was used to determine transfer efficacy of proteins. The membranes were incubated overnight in ADAM_12_ primary antibody (1:5000; Abcam, England, UK). For detection, horseradish peroxidase coupled secondary antibody (1:4000; Amersham Life Sciences, Sandton, JHB, South Africa) was used. Antigen-antibody complexes were visualized using ECL detection reagent (Amersham Life Sciences, Sandton, JHB, South Africa) and exposed to an autoradiography film (Hyperfilm ECL, RPN 2103) and light emission was detected. All films were analyzed by means of densitometry (UN-SCAN-IT; Silk Scientific Inc., Utah, UT, USA) and normalized data expressed in arbitrary units (AU). In all instances the membranes were stripped, by incubating in 0.2 M NaOH and reblotted with antibody against β-tubulin (1:1000; Cell Signalling Technology, Beverley, MA, USA) to verify the uniformity of protein load across the test samples.

### 2.7. Statistical Analysis

All data are presented as mean ± standard error of the mean (SEM). Statistical significance was analyzed by a two-way ANOVA, followed by a Bonferroni *post hoc* test. *p < 0.05* was considered as statistically significant. Statistical analysis of data was performed using GraphPad Prism version 5.

## 3. Results

### 3.1. Chronic P. Glandulosa Treatment Accelerates Repair of Muscle Ultrastructure

**Figure 1:**
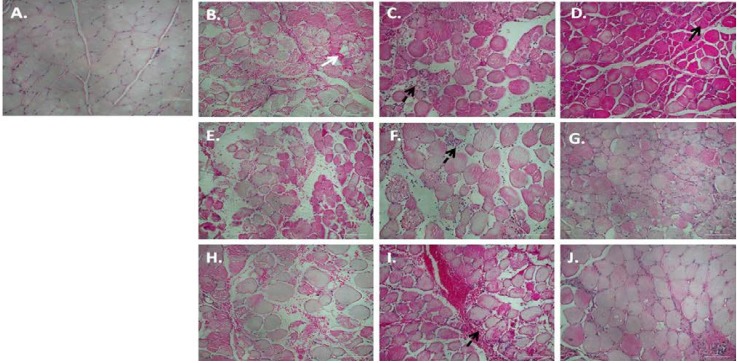

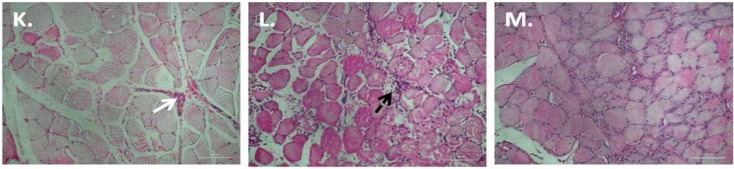
Ultrastructural changes over time after experimental contusion injury, stained by H & E. Figure (**A**) represent uninjured samples; Figures (**B**–**D**) represent samples taken from PLA animals at 3 h (**B**), 1 day (**C**) and 7 days (**D**) post-injury; Figures (**E**–**G**), (**H**–**J**) and (**K**–**M**) represent similar time points in the PG-CHR, PG-AI and NSAID groups respectively. Scale bar represents 100 μm; Figures (**B**), (**E**), (**H**) and (**K**) represent muscle fiber destruction and vascular disruption; Immune cells infiltration into the injured area is visible from 1 day post-injury (Figures (**C**), (**F**), (**I**) and (**L**)). *Solid white arrows* indicate red blood cells, *solid black arrows* points to newly regenerated muscle fibers and *dashed arrows* indicate immune cells.

Qualitative microscopic analysis of the fiber architecture post-contusion injury indicated that irrespective of treatment, the blunt force to the muscle belly significantly damaged and disrupted the skeletal muscle fibers, resulting in red blood cell accumulation in the interstitial spaces at 1 h and 3 h after injury (Figure 1B,E,H,K). Representative pictures for 1 h after injury are not included as differences between 1 and 3 h post-injury cannot be easily discerned visually. Edema was present in both treated and untreated groups, confirmed by the widening of the interstitial spaces between the fibers at this early time point. Histological comparison between the placebo (PLA) and the treatment groups illustrated a significant influx of immune cells 1 day after injury in all four groups. However, this influx was relatively limited in the group chronically treated with *P. glandulosa* (PG-CHR), when compared to all other groups. The immune cells remained visible in the injured area of the PLA, post-injury treated group (PG-AI) and the non-steroidal anti-inflammatory treated (NSAID) groups for up to day 7 post-injury (Figure 1D,J,M), but were undetectable in the PG-CHR group at the same time point (Figure 1G). This significant reduction in infiltrating neutrophils in the chronically treated *P. glandulosa* group is also reflected in the immunohistochemistry slides (Supplementary data: Addendum B, Figure 1C,F). By day 7 only the chronically treated *P. glandulosa* group (Figure 1G) displayed near normal muscle architecture, indicative of successful progressing muscle regeneration.

### 3.2. P. Glandulosa Treatment Blunted the Neutrophil Response to Contusion Injury

**Figure 2:**
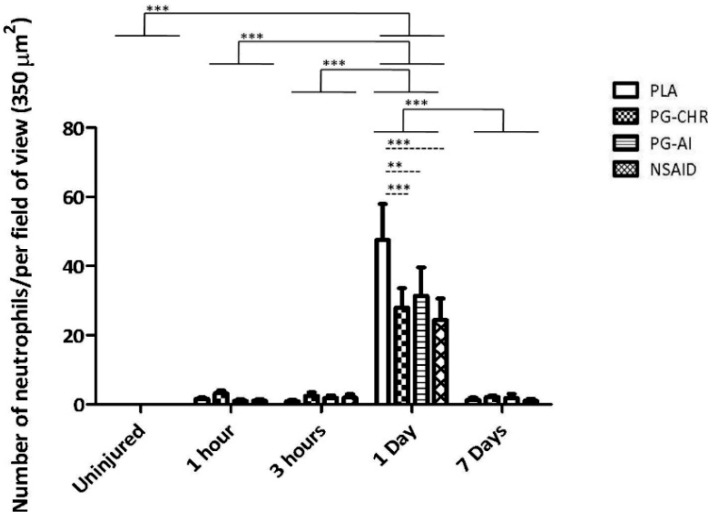
Neutrophil (His48 stain) infiltration into injured area after contusion injury with/without *P. glandulosa* treatment. The data are expressed as mean ± SEM. Analysis was done by two-way ANOVA. *n* = 5 per time-point/per group; Differences over time are indicated by solid lines and broken black lines indicate group differences at specific time points; Significance: All groups: *** *p <* 0.0001 uninjured *vs.* 1 day; 1 h *vs.* 1 day; 3 h *vs.* 1 day; 1 day *vs.* 7 days. 1 Day: *** *p <* 0.0001 PLA *vs.* PG-CHR; PLA *vs.* NSAID; ** *p <* 0.05 PLA *vs.* PG-AI.

Clear differences were evident between the various experimental groups with regards to neutrophil infiltration. No neutrophils were present in the any of the experimental groups before injury, whereas contusion injury resulted in a significant (between 30- and 40-fold) transient elevation in neutrophils on day 1 after injury, which normalised by day 7 post-injury (*p <* 0.0001) (Figure 2). On day 1 post-injury, the PG-CHR (*p <* 0.0001), PG-AI (*p <* 0.001) as well as the NSAID treatment groups (*p <* 0.0001) displayed a significantly lower number of neutrophils compared to the untreated group (PLA). Furthermore, the magnitude of the neutrophil response as assessed on day 1 post-injury was similar in these three treatments groups.

### 3.3. P. Glandulosa Treatment Did Not Affect Macrophage Response to Contusion Injury

**Figure 3:**
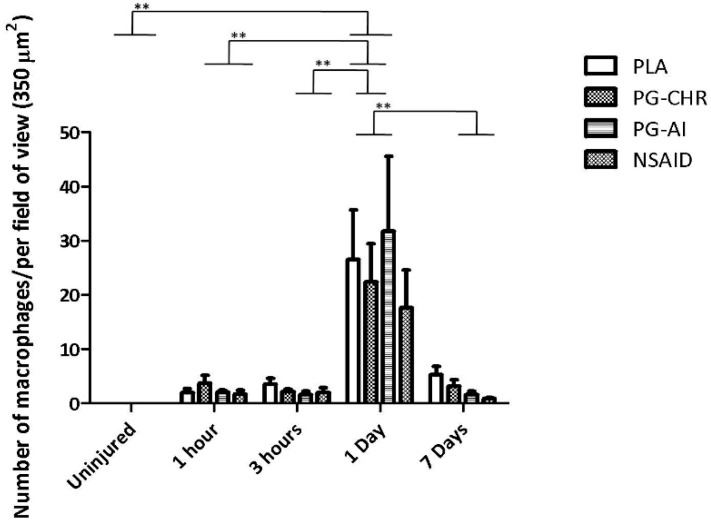
Macrophage infiltration into injured area after contusion injury with/without *P. glandulosa* treatment. The data are expressed as mean ± SEM. Analysis was done by two-way ANOVA. *n* = 5 per time-point/per group; Differences over time are indicated by solid lines; Significance: All groups: **p < 0.001 uninjured *vs.* 1 day; 1 h *vs.* 1 day; 3 h *vs.* 1 day; 7 days *vs.* 1 day.

Similar to the neutrophil data, the presence of macrophages was undetectable in the uninjured control samples (Figure 3). Of the time-points assessed, the peak number of macrophages (*p <* 0.001) present in the injured area, was 1 day after injury in all four experimental groups. These increased values had again normalised by day 7 after injury (*p <* 0.001). None of the treatments seem to have any effect on macrophage infiltration at the time points assessed.

### 3.4. ADAM_12_ Expression is Enhanced in Response to Chronic P. Glandulosa Treatment

**Figure 4:**
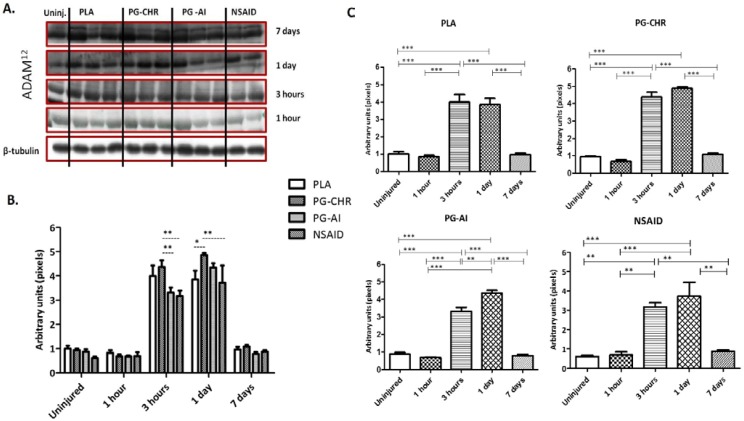
ADAM_12_ expression in skeletal muscle following a contusion injury. (**A**) Representative Western blots. Top 4 bands represent ADAM_12_ expression and the bottom bands represent β-tubulin expression (confirm equal loading of the protein); (**B**) Combined data for all the different groups; (**C**) Statistical differences observed between the different time-points in each experimental group. Values are expressed relative to the uninjured values. The data are expressed as mean ± SEM; Analysis by two-way ANOVA; *n* = 5 per time-point/per group; Differences over time are indicated by solid lines and broken black lines indicate group differences at specific time points; Significance: *** *p <* 0.0001, ** *p <* 0.001 and * *p <* 0.05.

According to the Western blot analysis, expression of the satellite cell proliferation marker, ADAM_12_, was significantly elevated from 3 h post-injury (*p <* 0.0001) and this significant elevation persisted for at least 24 h (*p <* 0.0001), with the expression again normalized to uninjured levels on day 7 after injury, in all experimental groups (*p <* 0.0001) (Figure 4A,B). Of all three treatments assessed, the 8-week chronic treatment with *P. glandulosa* (PG-CHR) showed the most significant effect with significantly increased (*p <* 0.05) expression of ADAM_12_, on day 1 post-injury, when compared to the PLA group. Although post-injury treatment (PG-AI) seemed to suppress ADAM_12_ expression at 3 h, when compared to PLA, it was associated with a significant increase in ADAM_12_ expression from 3 to 1 day. NSAID treatment was associated with a similarly suppressed ADAM_12_ expression at 3 h, but with this treatment, the relative suppression persisted at 1 day after injury. Indeed, the NSAID group expressed lower levels of ADAM_12_, compared to the chronically treated *P. glandulosa* group at both 3 h and 1 day post-injury, significantly so on the latter (*p <* 0.001). For the sake of clarity the statistical differences observed between the different time-points in each experimental group is illustrated in Figure 4C.

### 3.5. Desmin Expression is Increased in Response to Chronic P. Glandulosa Treatment

**Figure 5:**
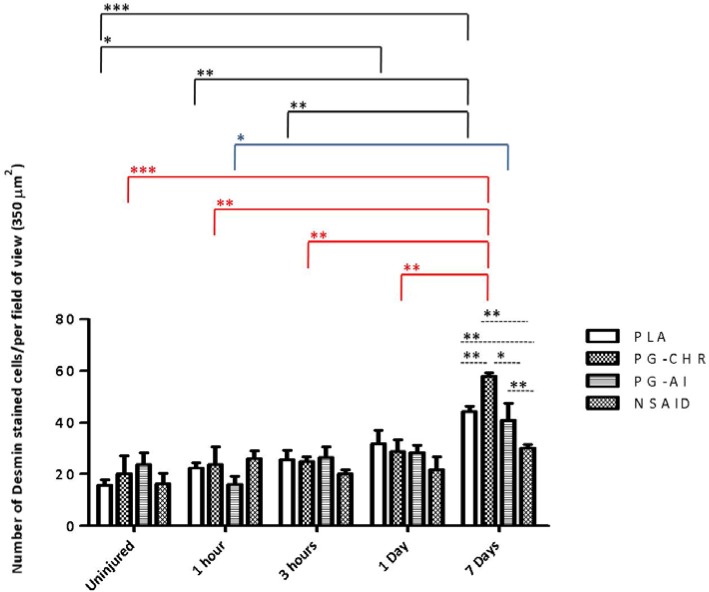
Desmin expression in skeletal muscle following a contusion injury. The data are expressed as mean ± SEM; Analysis by two-way ANOVA; *n* = 5 per time-point/per group; Differences over time are indicated by solid black lines (PLA), solid blue lines (PG-CHR), solid red lines (PG-AI) and broken black lines indicate group differences at specific time points; Significance: *** *p <* 0.0001, ** *p <* 0.001 and * *p <* 0.05.

Desmin expression was found to steadily increase after injury, with highest values at the 7 days post-injury time point, in all four different experimental groups. At the 7-day post-injury time-point, the chronically treated *P. glandulosa* group (PG-CHR) displayed significantly elevated desmin expression compared to all other groups (Figure 5). While post-injury *P. glandulosa*-treatment had no effect on the expression of desmin, the NSAID-treated group displayed significantly decreased desmin expression, when compared to all other groups, indicative of delayed regeneration.

## 4. Discussion

When muscle is injured, such as during a contusion injury, chemotactic factors are released by the myocytes and other surrounding cells, which results in immune cell mobilization and attraction to the injured area [3,9,31]. In this study we focused on *P. glandulosa* as a possible pre- and/or post-injury treatment option after a contusion injury. We assessed effects on neutrophil and macrophage infiltration into the injured area, as well as associated consequences in the context of regeneration.

Indeed, we present compelling evidence for an effect of *P. glandulosa* at tissue level in the early response to muscle injury. Firstly, chronic *P. glandulosa* treatment was found to significantly reduce neutrophil infiltration into the injured area, suggestive of a decreased pro-inflammatory signal and probably less neutrophil-associated secondary damage. Secondly, in support of this interpretation, we report an associated significant increase in the expression of ADAM_12_ (day 1 post-injury) and desmin (day 7 post-injury) suggesting an enhanced regenerative process. Since direct effects on satellite cell recruitment and incorporation into injured fibers were not assessed, no conclusion can be made on potential direct effects on this cell type. However, given the recent report of other plant products that had effects on both inflammation and satellite cell action directly after chronic treatment [7,24], this possibility cannot be excluded and remains to be elucidated. The fact that post-injury treatment of *P. glandulosa* did not yield similar benefits has more than one potential explanation. Firstly, the first post-injury dose may have been administered too late to affect early initiation steps for inflammation. However, since the neutrophil response was indeed significantly inhibited, this is unlikely. Rather, the active substance(s) also target other, as yet unknown, cellular targets for which modulation may occur over a longer time-period, rather than acutely.

The current study is the first to illustrate potential benefit for *P. glandulosa* in the context of muscle injury and inflammation. As a result, the potential mechanism(s) for the effects reported has not yet been extensively investigated. Neutrophil blunting effects have been reported previously, albeit with other natural substances [7,32,33,34]. From the literature, the ability of *P. glandulosa* to blunt neutrophil infiltration into the site of injury suggests that treatment either caused: (i) a lesser degree of activation of these immune cells; (ii) a lesser capacity for extravasation of the neutrophils; or (iii) decreased maturation and proliferation of neutrophils. Plant-derivatives have previously been shown to effectively reduce the infiltration of neutrophils into the site of injury by either reducing neutrophil extravasation from the blood [24], blunting neutrophil migration [24], partially inhibiting the activation of the immune cells [33] or inhibiting neutrophil chemotaxis [34]. A limitation of the current study was that time-points between 1 and 7 days post-injury were not assessed. Since macrophages usually peak around day 5 post-injury, we may have missed macrophage responses to the treatment, which most likely occurred 5 days after injury. Nevertheless, we show promising beneficial effects on inflammation in the context of neutrophils, so that more product-specific research is warranted, to enable firmer conclusions on mechanisms.

Furthermore, many phytomedicines exert effects through antioxidant mechanisms. Earlier findings have demonstrated that neutrophil attraction, adhesion and migration can be influenced by an increase in ROS generation [35] and that antioxidant treatment, which scavenges ROS, would alleviate the excessive ROS production and decrease attraction, adhesion and migration of circulating neutrophils [36,37]. However, in a previous study, conducted in our laboratory (unpublished data), the antioxidant capacity of *P. glandulosa* was evaluated in terms of lipid hydroperoxides (LOOH) and thiobarbituric acid reactive substance (TBARS) assays and we could find nosignificant differences between control and treated groups. It therefore seems reasonable to assume that *P. glandulosa* treatment did not act as an antioxidant.

In addition to the beneficial effects of the experimental product assessed, our results illustrate some alarming undesired effects of the NSAID, diclofenac. Similar to previous findings [38,39,40], NSAID treatment immediately after injury, resulted in the blunting of the inflammatory phase, by decreasing neutrophil infiltration into the injured area (Figure 2). In this context, and in the light of the rather inconclusive macrophage data, the natural and NSAID treatments seem similar in action. However, when turning to other parameters assessed, large differences become evident.

The level of ADAM_12_ expression in gastrocnemius muscles, at different time-points after injury, has not been measured in an *in vivo* model before and is a novel aspect of the current study. Our results confirm that previous reports of low ADAM_12_ expression levels *in vitro* differentiated muscle fibers [41,42,43], with higher expression only during proliferation and very early stages of differentiation [44], can be extrapolated to the *in vivo* situation. Our data showing an increased ADAM_12_ response at 3 h post-injury is in accordance with previous research which documented that the first signs of myogenic differentiation occur between 3 and 8 h after injury [45]. It has been reported that myoblasts fuse to form myotubes and reconstruct damaged myofibers as early as 2 days after injury [46,47,48]. This data also coincides with our ADAM_12_ data showing significant elevation in ADAM_12_ expression 1 day after injury in PG-CHR, relative to PLA. Furthermore, in our study, at day 7 after injury, the expression of ADAM_12_ was reduced to basal levels, suggesting that most satellite cells have differentiated and fused to form differentiated myotubes by day 7. The histology (Figure 1) and desmin (Figure 5) data supports this interpretation. The fact that chronic *P. glandulosa* treatment increased the ADAM_12_ expression at early time-points relative to the PLA indicates that the treatment may facilitate more effective recovery by enhancing early proliferation. In stark contrast, the NSAID treatment resulted in significantly suppressed ADAM_12_ expression (particularly at 3 h), pointing to an inhibitory effect on repair. This undesired effect was also evident from the desmin response.

Desmin levels usually increase significantly during myogenesis and remain elevated in newly matured myofibers [49], which explains the relative late response in our protocol time course. On day 7, and in accordance with our other data suggesting more effective muscle fiber repair, desmin expression was significantly higher after chronic *P. glandulosa* treatment. In contrast, desmin expression was significantly lower than PLA after NSAID treatment, again pointing to an inhibitory effect of NSAID on repair, as suggested in the literature [3]. It is important to note that NSAID treatment was administered for up to 7 days in the current study. If NSAID administration could be limited to 1–2 days post-injury, it is possible that the initial suppression of repair could be overcome, while maintaining the beneficial effect of neutrophil blunting (and thus secondary damage) reported here. However, it is clear that NSAID treatment should not be administered after these very early time points post-injury.

## 5. Conclusions

Our data indicate that *P. glandulosa* treatment results in more effective muscle repair after sterile contusion injury–at least in part due to modulating effects on neutrophil infiltration–while longer term application of topical NSAID (diclofenac) may inhibit repair, resulting in ineffective recovery.

The fact that *P. glandulosa* is categorized as an invader tree in South Africa–and probably other countries of export too–illustrates the ethnopharmacological significance of our novel results: this tree seems to be an ideal candidate for harvesting for natural medicine, as there is no imminent risk of depleting natural resources of the plant. The use of an economical, natural and readily available substance, such as the one we identified here, as treatment to limit acute inflammation and increase muscle regeneration, could have far-reaching implications in not only the sporting arena and health sectors, but also in plant and wildlife conservation.

## Supplementary Files

Supplementary File 1

Supplementary File 2
