# Supplementary material for: Chronic Prosopis Glandulosa Treatment Blunts Neutrophil Infiltration and Enhances Muscle Repair after Contusion Injury
Source: Nutrients. 2015 Jan 23;7(2):815–30. doi: 10.3390/nu7020815 (PMC4344562; doi:10.3390/nu7020815)
Supplement: Supplementary File 1 [file nutrients-07-00815-s001.pdf]

J. MULLER

LABORATORIES (PTY) LTD
Reg. No.1980/004037/07
ANALYTICAL CHEMISTS

P.O. BOX 511
PAARDEN EILAND 7420
REP. OF SOUTH AFRICA
TELEPHONE: 27-021-5118301/2
FAX: 27-021-5103800
E-mail: jmlabs@iafrica.com

SANAS
ACCREDITED LABORATORY

OFFICE & LABORATORIES AT:
30 MARINE DRIVE
PAARDEN EILAND 7405
REP. OF SOUTH AFRICA

Our Ref LN605879-8C-09

No T.0054
Test Report

Date of Issue: 30 JUNE 2006

Certificate of Analysis

This is to certify that the sample listed below was analysed

SUBMITTED BY: CONBRIO BRANDS
P.O. BOX 291
CENTURY CITY
7446
ATTENTION: G DEYSEL

SAMPLE TYPE: PLANT MATERIAL (DIABETIC SUPPLEMENT)

SAMPLE MARKS: "DIAVITE"

**DATE SAMPLE RECEIVED:**	05 MAY 2006
**DATE ANALYSIS STARTED:**	12 MAY 2006
**DATE ANALYSIS COMPLETED:**	30 JUNE 2006

Analysis relates only to the sample/s tested:

SEE ATTACHED PAGE 2

LABORATORY MANAGER
C VISSER
APPROVED SIGNATORY
