# Supplementary material for: Chronic Prosopis Glandulosa Treatment Blunts Neutrophil Infiltration and Enhances Muscle Repair after Contusion Injury
Source: Nutrients. 2015 Jan 23;7(2):815–30. doi: 10.3390/nu7020815 (PMC4344562; doi:10.3390/nu7020815)
Supplement: Supplementary File 2 [file nutrients-07-00815-s002.pdf]

OFFICIAL METHODS OF ANALYSIS OF AOAC INTERNATIONAL 17TH EDITION REVISION #2, 2003

		g/100g
*QALNITRO (Ref AOAC 984.13)	Protein (N x 6,25)	11,4
*QALDF (Ref AOAC 985.29)	Dietary Fibre	21,6
*QALFAT (acid hydrolysis)	Fat	2,62
AOAC 971.18	Fructose	4,12
AOAC 971.18	Glucose	0,89
AOAC 971.18	Sucrose	<0,02
AOAC 971.18	Sorbitol	<0,02
AOAC 971.18	Maltose	30,9
AOAC 971.18	Total sugars	35,9
AOAC 950.46	Moisture	7,14
AOAC 942.05	Ash	4,60
By difference	Carbohydrates	52,6
		mg/100g
AOAC 968.08	Sodium (Na)	37
AOAC 968.08	Potassium (K)	1475
AOAC 968.08	Calcium (Ca)	172
AOAC 968.08	Magnesium (Mg)	114
AOAC 968.08	Zinc (Zn)	1,2
AOAC 968.08	Iron (Fe)	1,7
		kJ/100g
	Energy Value	1133
		μg/kg
AOAC 990.33	Aflatoxin	
	B_1	<2
	B_2	<2
	G_1	<2
	G_2	<2

Values reported as less than (<) are lower than the limit of detection for the method employed.

*** Tested in accordance with SANAS accredited test methods**
Analysis not marked with an asterisk, are not included in the SANAS Schedule of Accreditation of this laboratory.

ANALYST
W vd MERWE

ANALYST
E NEETHLING

ANALYST
A BOTHA

LAB TECHNICIAN
S THERON

ANALYST
C VISSER

ANALYST
S TERRY

LABORATORY MANAGER
C VISSER
APPROVED SIGNATORY
